# Risk factors of kidney stone disease: a cross-sectional study in the southeast of Iran

**DOI:** 10.1186/s12894-021-00905-5

**Published:** 2021-10-08

**Authors:** Parvin Khalili, Zahra Jamali, Tabandeh Sadeghi, Ali Esmaeili-nadimi, Maryam Mohamadi, Amir Moghadam-Ahmadi, Fatemeh Ayoobi, Alireza Nazari

**Affiliations:** 1 Department of Epidemiology, School of Public Health, Social Determinants of Health Research Centre, Rafsanjan University of Medical Sciences, Rafsanjan, Iran; 2 Non-Communicable Diseases Research Center, Rafsanjan University of Medical Sciences, Rafsanjan, Iran; 3 Department of Pediatric Nursing, School of Nursing and Midwifery, Non-Communicable Diseases Research Center, Rafsanjan University of Medical Sciences, Rafsanjan, Iran; 4 Occupational Safety and Health Research Center, NICICO, World Safety Organization and Rafsanjan University of Medical Sciences, Rafsanjan, Iran; 5 Department of Neurology, School of Medicine, Non-Communicable Diseases Research Center, Rafsanjan University of Medical Sciences, Rafsanjan, Iran

**Keywords:** Kidney stone, Risk factors, Prospective epidemiological research studies in Iran (PERSIAN)

## Abstract

**Background:**

The prevalence of kidney stones in the world is increasing and environmental factors seem to play a major role in this issue. The aim of the present study was to investigate the prevalence of risk factors of kidney stones in the adult population of Rafsanjan city based on the data of the Rafsanjan Cohort Study (RCS).

**Methods:**

In the baseline phase of this study, 10,000 people aged 35 to 70 years are enrolled in the RCS, as one of the prospective epidemiological research studies in Iran. From this population, 9932 participants completed related demographic questionnaires as well as reported a history of diabetes mellitus, kidney stone, and hypertension diseases. The obtained data were analyzed using univariable and multivariable logistics regression.

**Results:**

According to the obtained results, 46.54% of the studied population were male and 53.46% were female. The mean age of the participants was 49.94 ± 9.56 years. 2392 people accounting for 24.08% of the population had kidney stones. After adjustment of the variables, six variables of gender, WSI, no consumption of purified water, BMI, and history of hypertension and diabetes were found to be significant related factors of kidney stone disease.

**Conclusions:**

Gender, hypertension, obesity, diabetes, and personal habits like alcohol consumption, opium use and, cigarette smoking are effective in the development of kidney stones. So, by identifying the susceptible patients and teaching them, the burden of the disease on society and the individual can be reduced. The results of this study are helpful to health care providers for preventive planning for kidney stone disease.

## Background

The prevalence and incidence of kidney stones is rising worldwide [1]. Kidney stones are hard deposits of minerals (calcium, oxalate and phosphate) which are formed from dissolved minerals in the urine and are usually excreted in the urethra [2]. Kidney stones are the third most common urinary tract problem after urinary tract infections and prostate disorders [3]. Kidney stones are classified into calcium oxalate, calcium phosphate, uric acid, cysteine, struvite, and mixed stones types, depending on the material of the stones. Calcium stones account for almost 70–80% of all kidney stones [4].

Risk factors related to kidney stones are different among different population groups and environmental factors have a key role in their pathogenesis [5]. Research on urological patients has shown that the incidence of kidney stones can be associated with sex, race, geographic region, occupation, hot climate, positive family history, unhealthy diet (excessive intake of caffeine, salt, dairy products, animal proteins and fat) [6], smoking, alcohol consumption, physical activity, obesity, low fluid intake, dehydration [2] socioeconomic status, education [5], water quality [7], high intake of vitamins D and C [8], genetic background [9] and comorbid metabolic disorders (diabetes mellitus, hypertension, chronic kidney disease, and cardiovascular disease) [1].

Epidemiologically, about 5% of women and 12% of men experience kidney stones during their lives [9]. Basiri et al*.* which conducted a study on the prevalence of urinary stones among 24 provinces of Iran, reported an average age of 41.5 years for the patients [7]. In another study, Safarinejad et al*.* found that the average cumulative recurrence of kidney stones was 16% after 1 year, 33% after 5 years and 53% after 10 years [5].

Iran is located on the kidney stone belt and this health issue is of high prevalence (5.7%) in this country [5, 10]. Kidney stone disease is a costly disease because it imposes a major financial burden to the patient and society [11]. Epidemiological evaluation of the issue, particularly assessment of the related risk factors, may result in finding appropriate approaches that reduce the risk of the development of kidney stones. Also, previous studies have shown that interventions in nutrition and water composition can prevent the occurrence of kidney stones [6, 12]. In some studies, contradictory results have been obtained about the risk factors associated with the formation of kidney stone [1, 2, 5–8], and additional studies should be performed to resolve the existing ambiguities. On the other hand, limited studies in Iran have reported demographic factors affecting the kidney stones and the prevalence of kidney stones among probabilistic samples in a population-based study. Accordingly, the present study was aimed to investigate the related factors of kidney stone disease in the adult population of Rafsanjan city, based on the data of the Rafsanjan Cohort study (RCS).

## Methods

### Study population

The population of this study was actually the population of the RCS, one of Prospective Epidemiological Research Studies in Iran (PERSIAN). In the RCS, it was designed to recruit a total of 10,000 participants of both genders aged 35–70 years from four pre-determined districts (2500 participants from each site) of Rafsanjan city (in the Southeast of Iran) including both urban and suburban areas. Of all the participants, 9932 had a complete medical questionnaire and entered our study using census sampling method. Those who had incomplete medical questionnaire met our exclusion criterion and so were excluded [13]. The following inclusion criteria were considered in the selection of the population: (1) inclusion of areas with minimum migration rates in order to limit loss to follow-up rate (2) inclusion of populations with different socio-economic levels, as well as environmental and occupational exposures [13]. The protocol of the present study was designed in accordance with the Persian cohort study and also was approved by the Ethics Committee of Rafsanjan University of Medical Sciences with the Ethical code of IR.RUMS.REC.1399.029.

### Outcome assessment

Expert interviewers interviewed each participant and completed the related questionnaires about his/her socioeconomic status, demography, occupational status, personal habits, history of disease, biochemical tests, blood pressure, physical activity and body mass index (BMI). All of the questionnaires were previously validated in the PERSIAN cohort study [14].

Based on the self-administered questionnaire, kidney stone, hypertension and diabetes was identified and included in the history of diseases if the disease was previously diagnosed by a doctor. Also, the expert interviewers asked questions about the signs of the diseases and the treatments used was overweight and obesity, intake of purified water, habits and lifestyle were also self-reported by the participants [13].

Education level was coded as (1) Illiterate, (2) Elementary, (3) Guidance school and Diploma, (4) College education [13]. It is noteworthy that the informed consent was obtained from the legal guardians of the illiterate participants.

The daily physical activity of the participants was weighted based on its relative metabolic cost, known as a metabolic equivalent (MET), and MET-h/day for 24 h is derived in this way [14, 15].

Data on the personal habit including cigarette and hookah smoking, alcohol drinking, and opium consumption were coded as yes (currently or formerly) and no (never) [13].

The wealth score index (WSI) was classified into four categories: low income (1st quartile: ≤ − 0.6069), low-middle income (2nd quartile: − 0.607 to 0.0349), middle-high income (3rd quartile: 0.035 to 1.169) and high income (4th quartile: ≥ 1.170) [14].

The source of drinking water was also questioned; whether purified water was consumed or not [13].

### Statistical analyses

Related factors for kidney stones including sociodemographic characteristics, life style and history of chronic disease were identified using relevant epidemiological texts and based on subject matter knowledge. For describing the distribution of the data, the frequency (percentage) of the categorical variables and the mean ± standard deviation of the quantitative variables were reported and their distribution among the case and control groups were compared using chi-square and independent t-test tests, respectively. The strength of the association between histories of kidney stones and relevant related factors was evaluated using odds ratios (ORs) and confidence intervals (CIs). We used univariable and multivariable logistics regression analyses to estimate odds ratios with 95% confidence intervals. For this purpose, separate models at bivariate level were run to obtain variables associated with kidney stone, at first. Afterwards, variables with a *p* value < 0.25 were included in the multivariable logistic regression model to calculate the adjusted odds ratio (OR) and 95% confidence interval (CI). The goodness-of-fit of the adjusted model was assessed using Hosmer–Lemeshow test. To clarify how kidney stone can be identified by gender, age, education level, WSI, cigarette smoking, alcohol drinking, opium consumption, hookah smoking, BMI, and history of chronic disease (diabetes and hypertension), receiver operating characteristic (ROC) curve analysis was applied. According to the method suggested by Swets [16] the area under the ROC curve follows: less accurate (0.5 < AUROC < 0.7), moderately accurate (0.7 < AUROC < 0.9), highly accurate (0.9 < AUROC < 1), and perfect tests (AUROC = 1). All of the analyses were performed using State V.12. All *p* values were two-sided, and *p* values < 0.05 and 95% confidence intervals were considered as statistically significant.

## Results

This study included 9932 participants, 4622 men (46.54%) and 5310 women (53.46%) with the mean age of 49.94 ± 9.56 years.

The details of the demographic characteristics including age, gender, education level, habits and WSI were presented in Table 1. As one can see, the distribution of these characteristics was different in the case (with kidney stone) and the control (without kidney stone) groups. In addition, we compared the distribution of lifestyle-related variables, as well as, history of diabetes and high blood pressure between the two groups.

**Table 1:** Baseline characteristics of the Rafsanjan cohort participants with and without kidney stone

Characteristics	Total (n = 9932)	Kidney stone (n = 2392)	No kidney stone (n = 7540)
Age—years (Mean ± SD)	49.94 ± 9.56	51.02 ± 9.48	49.59 ± 9.56
Age—no. (%)			
35–44	3433 (34.57)	725 (30.31)	2708 (35.92)
45–54	3028 (30.49)	723 (30.23)	2305 (30.57)
55–64	2745 (27.64)	735 (30.73)	2010 (26.66)
≥ 65	726 (7.31)	209 (8.74)	517 (6.86)
Gender—no. (%)			
Female	5310 (53.46)	1132 (47.32)	4178 (55.41)
Male	4622 (46.54)	1260 (52.68)	3362 (44.59)
Education—no. (%)			
Illiterate	948 (9.55)	245 (10.24)	703 (9.33)
Elementary	2537 (25.56)	640 (26.76)	1897 (25.18)
Guidance school and Diploma	4820 (48.55)	1102 (46.07)	3718 (49.34)
College	1622 (16.34)	405 (16.93)	1217 (16.15)
Physical activity (mean ± SD)	38.79 ± 6.32	38.85 ± 6.30	38.77 ± 6.33
Alcohol consumption—no. (%)			
Yes	1352 (13.64)	361 (15.13)	991 (13.17)
No	8599 (86.36)	2025 (84.87)	6534 (86.83)
Cigarette smoking—no. (%)			
Yes	2542 (25.59)	628 (26.25)	1914 (25.38)
No	7390 (74.41)	1764 (73.75)	5626 (74.62)
Hookah smoking—no. (%)			
Yes	1718 (17.30)	466 (19.48)	1252 (16.60)
No	8214 (82.70)	1926 (80.52)	6288 (83.40)
Opium consumption—no. (%)			
Yes	2378 (23.94)	616 (25.75)	1762 (23.37)
No	7554 (76.06)	1776 (74.25)	5778 (76.63)
Drinking of purified water in lifetime—no. (%)			
Yes	4692 (47.47)	1005 (42.16)	3687 (49.16)
No	5192 (52.53)	1379 (57.84)	3813 (50.84)
Hypertension—no. (%)			
Yes	2235 (22.50)	668 (27.93)	1567 (20.78)
No	7697 (77.50)	1724 (72.07)	5973 (79.22)
BMI—no. (%)			
< 25	2865 (28.87)	610 (25.51)	2255 (29.94)
25–29.9	4069 (41.01)	1025 (42.87)	3044 (40.41)
≥ 30	2989 (30.12)	756 (31.62)	2233 (29.65)
Diabetes mellitus—no. (%)			
Yes	1933 (19.46)	576 (24.08)	1357 (18.00)
No	7999 (80.54)	1816 (75.92)	6183 (82.00)
WSI—no. (%)			
Low	2720 (27.41)	600 (25.09)	2120 (28.15)
Low-middle	2462 (24.81)	608 (25.43)	1854 (24.62)
Middle-high	2780 (28.02)	720 (30.11)	2060 (27.35)
High	1960 (19.75)	463 (19.36)	1497 (19.88)

*BMI* body mass index, *WSI* wealth score index

The prevalence of kidney stone was associated with alcohol drinking, opium consumption, and hookah smoking. Moreover, kidney stone was more prevalent in the people with middle-high WSI, high BMI, and with a history of diabetes and blood pressure.

Majority of the case group (people with kidney stone) was male and had significantly higher alcohol consumption, higher opium use, Hookah smoking and middle to higher WSI in comparison to the control group (people without kidney stone). Consumption of purified drinking water was considerably lower in the case group compared with the control group. In addition, the cases were more likely to have a high BMI, history of diabetes and high blood pressure compared with the study controls (Table 1).

Table 2 represents the association of patient factors and kidney stone, using the univariate and multivariate analyses. The related factors associated with kidney stone in Univariate analysis were also assessed and the subjects with kidney stone were compared with the normal subjects (Table 2). The odds of having kidney stone were estimated for twelve factors: gender, age, education level, WSI, cigarette smoking, alcohol drinking, opium consumption, hookah smoking, BMI, consumption of purified water in lifetime, and history of chronic disease (diabetes and hypertension) (Table 2). It was found that all of these factors with the exception of cigarette smoking were significantly associated with kidney stone in the Univariate analysis.

**Table 2:** Patient factors and the odds of having kidney stone (n = 9932)

Patient factors	Univariable	Multivariable
	OR (95% CI)	OR (95% CI)
Age—year		
35–44	1	1
45–54	1.17 (1.04–1.32)	1.04 (0.92–1.19)
55–64	1.37 (1.21–1.54)	1.10 (0.95–1.28)
≥ 65	1.51 (1.26–1.82)	1.15 (0.93–1.42)
Gender		
Female	1	1
Male	1.38 (1.26–1.52)	1.57 (1.39–1.76)
Alcohol consumption		
No	1	1
Yes	1.18 (1.03–1.34)	1.12 (0.96–1.31)
Cigarette smoking		
No	1	1
Yes	1.05 (0.94–1.16)	0.80 (0.70–0.93)
Hookah smoking		
No	1	1
Yes	1.22 (1.08–1.37)	1.16 (1.02–1.32)
Opium consumption		
No	1	1
Yes	1.14 (1.02–1.26)	1.02 (0.88–1.17)
Drinking of purified water in lifetime		
Yes	1	1
No	1.33 (1.21–1.46)	1.20 (1.08–1.35)
Hypertension		
No	1	1
Yes	1.48 (1.33–1.64)	1.33 (1.18–1.50)
BMI		
< 25	1	1
25–29.9	1.24 (1.11–1.40)	1.24 (1.11–1.40)
≥ 30	1.25 (1.11–1.41)	1.30 (1.14–1.48)
Diabetes mellitus		
No	1	1
Yes	1.45 (1.29–1.61)	1.26 (1.12–1.43)
WSI		
Low	1	1
Low-middle	1.16 (1.02–1.32)	1.14 (1.01–1.30)
Middle-high	1.23 (1.09–1.40)	1.25 (1.10–1.42)
High	1.09 (0.95–1.25)	1.08 (0.94–1.25)

*BMI* body mass index, *WSI* wealth score index

**Fig. 1:**
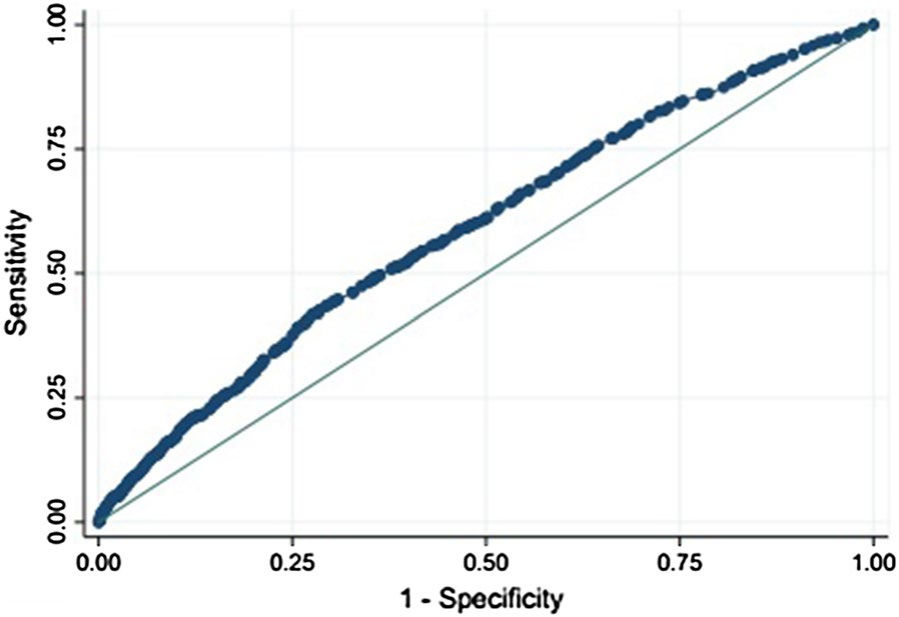
ROC curve of the related factors for the identification of kidney stone

After adjustment, only six variables of gender, 3 levels of the WSI (low, low-middle and middle-high), consumption of purified water in lifetime, BMI, history of hypertension and diabetes were shown to be significantly associated with kidney stone (Table 2). In the crude regression model, the odds of kidney stone was higher (odds ratio (OR): 1.38, 95%CI 1.26 to 1.52) among men compared with women and the odds increased after adjustment for confounders (adjusted model) (OR: 1.57, 95% CI 1.39 to1.76). In the crude regression model, the odds of kidney stone was slightly higher for all categories of WSI except high (low-middle OR: 1.16, 95% CI 1.02 to 1.32 and middle-high OR: 1.23, 95% CI 1.09 to 1.40) and this association persisted after adjustment for the confounders (low-middle OR: 1.14, 95% CI 1.01 to 1.30 and middle-high OR: 1.25, 95% CI 1.10 to 1.42). Also, in the crude regression model the odds of kidney stone were slightly higher in overweight and obese people (25 to 29.9 kg/m^2^ OR: 1.24, 95% CI 1.11 to 1.40 and ≥ 30 kg/m^2^ OR: 1.25, 95% CI 1.14 to 1.41), in comparison to normal-weight people and this association persisted after adjustment for the confounders (25 to 29.9 kg/m^2^ OR: 1.24, 95% CI 1.11 to 1.40 and ≥ 30 kg/m^2^ OR: 1.30, 95% CI 1.14 to 1.48). Regarding history of high blood pressure and diabetes, in the crude regression model, the odds of kidney stone was higher in people with history of high blood pressure (OR: 1.48, 95% CI 1.33 to 1.64) and diabetes (OR: 1.45, 95% CI 1.29 to1.61), in comparison to healthy people. Although, the odds of kidney stone was slightly reduced after adjustment for the confounders, the association persisted for high blood pressure (OR: 1.33, 95% CI 1.18 to 1.50) and diabetes (OR: 1.26, 95% CI 1.12 to1.43). In addition, no consumption of purified water in lifetime was associated with kidney stones (OR: 1.20, 95% CI 1.08 to 1.35) after adjustment for the confounders. Assessment of goodness-of-fit of the adjusted model using Hosmer–Lemeshow test showed that the model fits well (*p* = 0. 0811). After adjustment, ROC curve analysis was also employed to clarify how kidney stone can be identified by the variables previously listed. According to the ROC analysis, the AUROC was obtained to be 0.59 (95% CI = 0.586–0.61), for the presence of six variables of gender, WSI, consumption of purified water in lifetime, BMI, history of hypertension and diabetes (Fig. 1).

## Discussion

According to the obtained results, significant differences were observed when comparing the case and the control groups based on some demographic variables. Our results showed the highest risk of kidney stone development for male. In contrast, Rafiei et al. reported that women were at the higher risk kidney stone development [8]. However, Curhan found that kidney stone was more prevalent among men in the United States [17]. Shirazi et al., also reported that the majority of cases of kidney stones in Tehran in Iran are men (66.5%) [18]. There are other similar studies in Iran and abroad that confirmed our finding [5, 19–21]. The effects of sex hormones (estrogens and androgens) on urinary oxalate and calcium can be responsible for the higher risk of kidney stone development in men. Estrogen prevents the formation of kidney stones by increasing the production of citric acid [22]. Therefore, recommendations in terms of diet and physical activity for the prevention of kidney stone development in men seem necessary and also, follow-up of them is suggested.

Based on our results, subjects in the case group had higher alcohol consumption (15.13% vs 13.17%), opium use (25.75% vs 23.37), hookah smoking (19.485 vs 16.60) and cigarette smoking (26.25% vs 25.38%) compared with the control group. In this regard, Ketabchi et al. reported higher rate of opium dependency among people with recurrent kidney stone, in Iran [23]. In another study by Nalini et al. [2] 36.03% of the patients with kidney stone had the habits of smoking and 41.59% of them had the habits of alcohol consumption, which are higher than our results. In consistence with the results of the present work, the study conducted by Tamadon et al. showed that the proportion of smokers in nephrolithiasis patients was significantly higher than normal subjects. The relation between cigarette smoking and the formation of kidney stone may be explained by the high levels of cadmium and lead in smokers’ body. Cigarette smoking may result in the induction of urolithiasis by reducing urinary flow and increasing the concentration of serum cadmium [2]. However, these associations were examined in the adjusted model and the obtained results showed that there was no association between cigarette smoking, alcohol consumption and opium use with kidney stones after adjusting for other related factors.

According to the results, cases were more likely to have a high WSI and BMI, history of diabetes and high blood pressure compared with the study controls. Even after adjustment for potential confounders, we found that high WSI and BMI, as well as, history of diabetes and high blood pressure increased odds of kidney stones. Similarly, Taylor et al. reported a positive correlation of the risk of kidney stones development with BMI and waist circumference (WC) [24]. Also, Nowfar et al. found that the incidence of nephrolithiasis was directly associated with obesity in both genders [25]. Moreover, the study of Curhan et al. revealed a positive association between the prevalence of calcium oxalate stone disease and BMI [17]. It can be proposed that, weight control and dietary interventions are preventive methods against the development kidney stones.

In our study, 27.93% of the case group had hypertension. In the study of Kalani et al. [26] 29.7% of patients with nephrolithiasis had hypertension, which is consistent with our results. Also, Shang et al. [27] showed that the risk of nephrolithiasis was directly associated with hypertension incidence. In addition, it has been shown that patients with hypertension as a related factor of the metabolic syndrome are more susceptible to kidney stones [5]. Accordingly, these findings are possibly of warning signs for these patients.

Our results showed that 24.08% of the cases had diabetes and the odds of kidney stone in diabetic cases was 1.26 compared with non-diabetic cases, after adjustment for the potential confounders. In this regard, Weinberg et al*.* [11] found that the severity of Type 2 diabetes was an important risk factor for kidney stone disease. Also Chung et al. reported that patients with kidney stones were at increased risk of diabetes after 5 years of follow-up [28]. However, Kabeya et al. [29] showed that there was no significant relationship between insulin resistance parameters and kidney stone risk, which is inconsistent with our study. Therefore, further studies are needed to clarify this possible relationship.

Based on the results of our study, odds of consumption of purified water in the case group was lower in comparison to the control group, and this parameter was a significantly association of kidney stone. In this regard and in contrast to our results, the results of a study by Basiri et al*.* on various capitals of Iran showed that the total hardness of tap water was not significantly associated with the regional prevalence of urinary calculus [7]. Also, Pubali et al. reported no considerable association between water quality and the occurrence of kidney stones [30]. Similarly our results, the results of the studies by Barkers and Donnan [31] and Churchill et al*.* [32] demonstrated a positive association between urinary calculus and the total hardness of drinking water.

Finally, ROC analyses revealed that variables of gender, WSI, no consumption of purified water in lifetime, BMI, and history of hypertension and diabetes are “less accurate” for the identification of kidney stones.

This study has strengths and limitations. One of the main strengths of our study is its population-based nature with a large sample size, and extensive data collection for the related factors of interest (e.g*.* age, sex, variables related to lifestyle and etc.). However, the study has some limitations too. For instance, cross-sectional design of the present study does not allow deriving any causal inferences. Or this fact that, cross-sectional studies are weak in predicting the risk factors of a model, because the temporality sequence between the related factors and kidney stones in these studies is not clear. In addition, it is possible that a number of the participants have not completely report the status of their kidney stones. On the other hand, there may have been some degrees of non-differential misclassification due to self-reporting and recall biases. Accordingly, it is suggested that the relationships reported here be reconsidered in the follow-up phase of the RCS. In addition, further research is recommended to determine the variables and to be able to suggest efficient ways to reduce the incidence of kidney stones in the general population.

## Conclusion

According to the results of the present study, factors such as gender, hypertension, obesity, diabetes and personal habits like alcohol consumption, opium use and cigarette smoking are related with the development of kidney stones. So, by identifying the susceptible patients and teaching them, the burden of the disease on society and the individual can be reduced. The results of this study are helpful to health care providers for preventive planning for kidney stone disease.

## Data Availability

The datasets used during the current study are available on the Persian Adult Cohort Study Center, Rafsanjan University of Medical Sciences, Iran. The data is not available publicly. However, upon a reasonable request, the data can be obtained from the authors.

## Abbreviations

RCS: The Rafsanjan Cohort Study
PERSIAN: Prospective epidemiological research studies in Iran
BMI: Body mass index
CIs: Confidence intervals
OR: Odds ratio
WC: Waist circumference
MET: Metabolic equivalent
WSI: Wealth score index
